# From Performance to Health: A Global Scientometric Analysis of the Evolution of CrossFit Research

**DOI:** 10.3390/sports14050213

**Published:** 2026-05-21

**Authors:** Gabriel de Souza Zanini, David Michel de Oliveira, Pedro Luiz Santorsula de Paula Oliveira, Eduarda Corteze Santos, Renata da Silva Alves Bolzam, Víctor Hernández-Beltrán, José M. Gamonales, Mário Cunha Espada, Danilo Alexandre Massini, Dalton Muller Pessôa Filho

**Affiliations:** 1 Department of Physical Education, School of Sciences (FC), São Paulo State University (UNESP), Bauru 17033-360, Brazildalton.pessoa-filho@unesp.br (D.M.P.F.); 2 Department of Physical Educaiton, Faculdades Integradas de Jahu (FIJ), Jau 17207-310, Brazil; 3 Graduate Program in Biosciences and One Health, Institute of Biosciences (IB), Federal University of Jataí (UFJ), Jataí 75801-615, Brazil; 4 Postgraduate Program in Human Development and Technology, São Paulo State University (UNESP), Rio Claro 13506-900, Brazil; dmassini@hotmail.com; 5 Graduate Program in Health Sciences, Institute of Health Sciences (ICS), Federal University of Jataí (UFJ), Jataí 75801-615, Brazil; 6 Training Optimization and Sports Performance Research Group (GOERD), Faculty of Sport Science, University of Extremadura, 10005 Cáceres, Spain; victorhb@unex.es (V.H.-B.); martingamonales@unex.es (J.M.G.); 7 Faculty of Education and Psychology, University of Extremadura, 06006 Badajoz, Spain; 8 Instituto Universitario de Investigación e Innovación en el Deporte (INIDE), University of Extremadura, 10003 Cáceres, Spain; 9 Escola Superior de Educação, Instituto Politécnico de Setúbal, 2914-504 Setúbal, Portugal; mario.espada@ese.ips.pt; 10 Sport Physical Activity and Health Research & Innovation Center (SPRINT), Sport Sciences School of Rio Maior (ESDRM), Instituto Politécnico Santarém, 2040-413 Rio Maior, Portugal; 11 Faculdade de Motricidade Humana, Centro Interdisciplinar de Performance Humana (CIPER), Universidade de Lisboa, 1499-002 Lisbon, Portugal; 12 Comprehensive Health Research Centre (CHRC), Universidade de Évora, 7000-645 Évora, Portugal

**Keywords:** CrossFit^®^, high-intensity functional training, scientometric analysis, sports science, exercise physiology

## Abstract

Scientific production on CrossFit^®^ has expanded alongside the growing popularity of the modality; however, multi-database scientometric analysis describing its structure, research trends, and knowledge gaps remains limited. Objective: This study conducted a scientometric analysis to identify patterns within the literature and to provide directions for future research. Methods: Searches were performed in the databases Web of Science, PubMed, and Scopus, including all publications available up to December 2024. The search identified 3927 records. After removing duplicates and excluding reviews, meta-analyses, and studies outside the scope, 526 original articles were included in the analysis. Scientometric analyses were conducted using Bibliometrix (version 4.3.2) implemented in R (version 4.4.2), with additional support from Microsoft Excel and VOSviewer (v1.6.20). Results: The results indicate a marked growth in publication output, with an average annual increase of approximately 37.5%, reflecting the increasing academic interest in the modality. The United States and Brazil emerged as leading contributors, supported by strong research infrastructure and expanding scientific communities. The thematic structure of the field is predominantly centered on physiological responses, performance outcomes, and injury-related topics, while psychosocial, behavioral, and population-specific dimensions remain comparatively underexplored. Despite the observed expansion, the findings suggest that quantitative growth has outpaced methodological diversification and longitudinal development within the field. In addition, a limited integration between scientific findings and applied training contexts was identified, highlighting a gap between research production and real-world practice. Conclusion: Overall, CrossFit^®^ research appears to be expanding toward a more diversified and structured scientific field; however, advancing the field will require greater methodological rigor, increased focus on longitudinal and integrative approaches, and stronger translation of scientific evidence into applied settings.

## 1. Introduction

CrossFit^®^ emerged in the United States in the early 2000s, developed by Greg Glassman, and has since been established as a conditioning program centered on high-intensity functional exercises that integrate Olympic weightlifting, cardiovascular training, and gymnastics. These elements are typically structured into sessions known as workouts of the day (WODs) [1,2]. Participation in CrossFit^®^ has been associated with improvements in overall physical fitness, cardiovascular capacity, and body composition [3], which are attributed to both the high energetic demands of the workouts and the endocrine–metabolic adaptations induced by high-intensity training [4].

In addition, the modality has been linked to psychological and social benefits, including enhanced motivation, greater adherence to exercise programs, and improved perceived well-being, effects often facilitated by the socially interactive and competitive environment inherent to CrossFit^®^ practice [5,6,7,8,9,10]. However, despite these reported benefits, the scientific literature remains heterogeneous in terms of methodological quality, and the scientific literature remains heterogeneous in terms of methodological approaches and study objectives, which may limit the comparability of findings across studies, limiting the strength of causal inferences.

On the other hand, the widespread dissemination of CrossFit^®^ is evidenced by its global expansion and the growing number of practitioners across diverse countries, particularly in the United States and Brazil [11,12]. This rapid growth has consolidated CrossFit^®^ as a widely adopted training strategy in both commercial fitness settings and structured conditioning programs aimed at health promotion and performance enhancement [13,14,15,16]. Furthermore, the modality has been implemented across multiple contexts, including recreational practice [9,10], competitive environments [17,18,19,20], and clinical or therapeutic applications [20,21,22]. This diversification underscores the importance of systematically understanding how scientific knowledge on CrossFit^®^ has evolved and how the field is structurally organized over time.

Scientific research on CrossFit^®^ has expanded substantially since its emergence as a training modality. Although frequently situated within the broader framework of High-Intensity Functional Training (HIFT), these constructs are not conceptually equivalent. CrossFit^®^ represents a proprietary training methodology characterized by standardized programming principles, formalized competitive structures, and a defined community-based training environment. In contrast, HIFT constitutes a broader scientific construct describing training modalities that involve constantly varied, functional movements performed at high intensity, which may or may not be directly associated with CrossFit^®^ practice. This distinction is particularly relevant in the scientific literature, where HIFT is often employed to generalize findings across heterogeneous training models [23,24]. However, such generalization may obscure meaningful differences in training structure, participant characteristics, and contextual factors. Accordingly, in the present study, CrossFit^®^ is treated as the primary unit of analysis, while HIFT is considered a broader conceptual framework to support interpretation when appropriate.

Previous investigations have attempted to characterize the development of this field. For instance, Feito et al. [25] conducted a content analysis of 104 studies published between 2007 and 2018, identifying a progressive increase in publications, a concentration of studies focused on physiological outcomes, a strong geographical concentration of research in the United States, and a limited number of clinical trials. Despite its contributions, that study was restricted to searches conducted in Google Scholar and PubMed and did not incorporate advanced scientometric approaches capable of capturing the structural dynamics of the literature. More recently, Stanciu and Voiculescu [26] performed a bibliometric analysis based on 296 articles retrieved exclusively from the Scopus database. Although their findings provided valuable insights into influential authors, leading countries, prominent journals, and recurrent keywords, the analysis remained limited to a single database and did not explore the conceptual structure or thematic evolution of the field.

Collectively, these limitations underscore the need for a more comprehensive scientometric investigation integrating multiple international databases and employing advanced bibliometric techniques to examine intellectual structures, collaboration networks, and thematic trends in CrossFit^®^ research. Such an approach enables a more robust understanding of the field’s development, facilitates the identification of emerging research directions, and reveals persistent gaps in the literature. Moreover, these insights may inform strategic academic decision-making, guide future research agendas, and support evidence-based practice within CrossFit^®^ training and related conditioning programs. Despite this expansion, the field has expanded rapidly, although important conceptual and methodological gaps remain insufficiently explored, with limited theoretical integration and relatively low emphasis on applied translation. This reinforces the need for a more critical and structured understanding of how CrossFit^®^ research is evolving beyond its initial exploratory phase.

In this context, the present study aimed to conduct a comprehensive scientometric analysis of the scientific literature on CrossFit^®^ training, describing its temporal evolution, the most productive authors, the most influential journals and documents, collaboration networks, and the geographical distribution of research across institutions and countries. Beyond mapping publication trends, this study seeks to provide a structural and conceptual interpretation of the evolution of CrossFit^®^ research, exploring how thematic organization, collaboration networks, and emerging research domains reflect broader developments in the scientific literature related to CrossFit^®^ and high-intensity training modalities.

## 2. Materials and Methods

### 2.1. Study Design

This study consisted of a scientometric review, a quantitative methodological approach used to analyze the dynamics of scientific production and to map the organization, structure, and evolution of knowledge domains [27]. Scientometric methods rely on bibliographic metadata extracted from scientific publications to identify patterns related to productivity, collaboration networks, citation structures, and thematic trends within a research field [28,29].

In the present study, only original research articles were included, as these documents represent the primary units of knowledge generation in the scientific literature [27,28]. Non-original documents, including narrative reviews, systematic reviews, editorials, conference abstracts, letters, and other forms of grey literature, were excluded because they do not present primary empirical data and may distort indicators of scientific productivity and citation impact in scientometric analyses [29].

### 2.2. Data Sources

The bibliographic databases Web of Science (WoS), PubMed, and Scopus were selected as data sources for this study. These databases were chosen due to their broad coverage of peer-reviewed journals, multidisciplinary scope, and the availability of structured bibliographic metadata suitable for scientometric analyses. In this line, these databases were selected due to their complementary coverage, indexing quality, and widespread use in bibliometric and scientometric research, allowing for a more comprehensive and methodologically robust dataset.

The integration of multiple databases was adopted to maximize sensitivity and reduce potential database-related bias, ensuring a more comprehensive identification of the scientific literature related to CrossFit^®^ training.

### 2.3. Time Frame

To comprehensively map the scientific production related to the topic, all publications indexed up to December 2024 were considered, without establishing a predefined retrospective time limit. The earliest article identified through the search strategy was published in 2012, reflecting the relatively recent emergence of CrossFit^®^ as both a training modality and a scientific research topic.

Including the entire available publication period allowed the present study to capture the early stages of knowledge development in this field and to track its subsequent growth and evolution over time.

### 2.4. Search Strategy

An initial exploratory phase was conducted to identify relevant descriptors and free-text terms associated with CrossFit^®^ research. During this stage, previously published studies indexed in the selected databases were examined in order to identify recurrent keywords appearing in article titles, abstracts, and author-defined keywords. These terms were then combined using Boolean operators (AND and OR) to optimize both sensitivity and specificity in the retrieval of relevant studies.

Following several iterative rounds of testing and refinement, a standardized and reproducible search strategy was developed and applied across the three databases. The final search string adopted in the present study was: (“CrossFit” OR “WOD” OR “HIFT” OR “functional fitness”) AND (“performance” OR “strength” OR “VO_2_max” OR “anaerobic” OR “nutrition” OR “diet” OR “supplement” OR “injury” OR “biomechanics” OR “motivation”). The final search string was selected after iterative testing, as it provided the highest sensitivity and the most comprehensive dataset for the purposes of this scientometric analysis.

Detailed information regarding the specific search syntax used in each database, including the platform configuration and the format of exported bibliographic records, is provided in the Supplementary Material (Table S1) to ensure transparency and reproducibility of the search procedure. The study followed general principles of systematic search transparency as recommended by PRISMA-S guidelines for reporting literature searches, although formal registration was not required due to the scientometric nature of the analysis.

The search strategy was applied in a standardized manner across the fields Title, Abstract, and Keywords in the three databases consulted. In PubMed, the search terms were retrieved from the Title/Abstract fields. In Scopus, the search was conducted in the TITLE-ABS-KEY field. In WoS, the search was performed using the Topic (TS) field, which includes Title, Abstract, Author Keywords, and Keywords Plus.

### 2.5. Data Collection

A total of 3927 records were initially retrieved from the selected databases: 914 from PubMed, 1517 from WoS, and 1496 from Scopus. The search results were exported in the native formats of each database, generating the files WebofScience.bib, PubMed.txt, and Scopus.bib. All bibliographic records were downloaded on 11 September 2025, ensuring a consistent temporal snapshot of the databases. To ensure consistency and reproducibility, the temporal scope of the analysis was restricted to studies published up to 31 December 2024. This approach allowed the establishment of a fixed and comparable time frame across all databases, minimizing potential biases related to database updating cycles and indexing delays.

Subsequently, the exported files were integrated into a single dataset for analysis. Data merging, standardization, and preliminary processing of bibliographic metadata were performed using Bibliometrix (version 4.3.2) implemented in R (version 4.4.2) [28,29]. This procedure allowed the harmonization of metadata fields across the three databases, enabling consistent downstream scientometric analyses.

### 2.6. Inclusion and Exclusion Criteria

The study included original research articles published up to 31 December 2024, regardless of publication language. The decision to include all languages aimed to minimize language bias and maximize the coverage of the scientific literature. The following non-original document types were excluded: narrative reviews, systematic reviews, meta-analyses, editorials, books, book chapters, technical notes, conference abstracts, letters to the editor, and grey literature. These exclusions were applied to ensure that the dataset consisted exclusively of primary research contributions, which represent the fundamental units of knowledge production in scientometric analyses.

### 2.7. Automated Screening

Automated screening procedures were conducted using Bibliometrix (version 4.3.2) implemented in R (version 4.4.2). During this stage, 1804 duplicate records were identified and removed, resulting in 2123 unique documents. In a subsequent automated filtering step, the software was used to identify and exclude non-original document types based on metadata fields (e.g., document type and publication category). This process resulted in the removal of 292 additional records, leaving 1831 articles eligible for the subsequent manual scope screening.

### 2.8. Manual Scope Screening

A subsequent manual scope screening was conducted to verify the thematic relevance of the remaining records. During this stage, 1305 documents were excluded for not meeting the predefined eligibility criteria. Specifically, studies were removed when they did not directly investigate CrossFit^®^ training or closely related high-intensity functional training contexts. The excluded records predominantly comprised: (i) general strength and conditioning programs unrelated to CrossFit^®^ or HIFT; (ii) studies focused exclusively on artistic gymnastics or other gymnastic disciplines without relevance to CrossFit^®^ practice; (iii) research centered on traditional resistance training, endurance training, or rehabilitation protocols not incorporating CrossFit^®^ methodologies; and (iv) publications addressing functional fitness solely at a conceptual level, without empirical evaluation of CrossFit^®^ training or comparable HIFT-based protocols.

Particular attention was devoted to distinguishing CrossFit^®^-specific research from broader HIFT or functional training studies. Articles were included when CrossFit^®^ was explicitly identified as the primary training modality or when the intervention clearly reflected CrossFit^®^-derived structures (e.g., WOD formats, benchmark workouts, or explicit methodological alignment). Studies employing the term HIFT were critically appraised and included only when their experimental design and contextual description demonstrated direct correspondence with CrossFit^®^-based training practices, consistent with criteria adopted in previous investigations [23,25,30,31,32]. For example, studies examining generalized circuit-based functional training without explicit methodological alignment with CrossFit^®^ principles or benchmark structures were excluded, whereas studies incorporating WOD-based protocols or explicitly describing CrossFit^®^-derived programming were retained. This strategy ensured the preservation of conceptual specificity while acknowledging the widespread use of HIFT as a scientific abstraction for CrossFit^®^-like training modalities.

**Figure 1:**
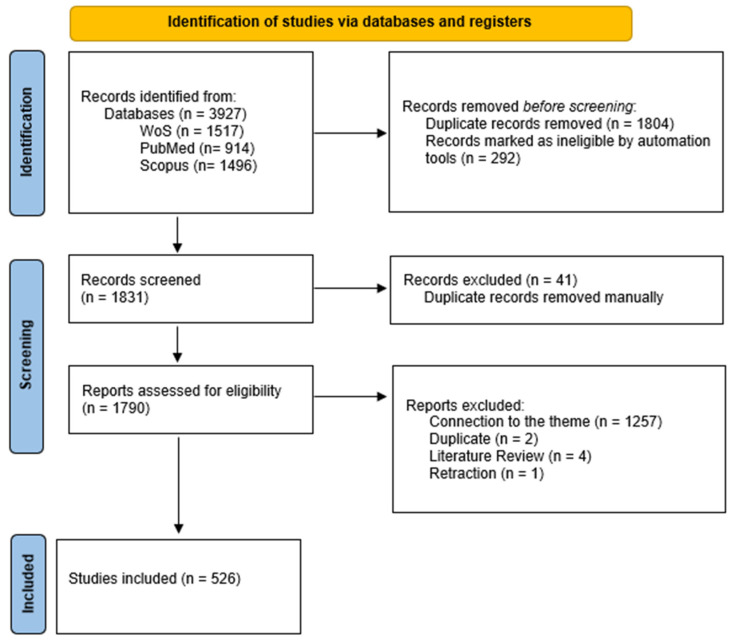
PRISMA-adapted flow diagram illustrating the identification, screening, eligibility, and inclusion stages of scientific articles related to CrossFit^®^ research. The diagram summarizes the database search results, duplicate removal, automated filtering of non-original documents, manual scope screening, and the final selection of studies included in the scientometric analysis.

In addition, a limited number of residual duplicates and non-original documents not identified during earlier screening stages were removed during manual verification. Following completion of this process, a final sample of 526 original research articles was retained for scientometric analysis (Figure 1). The screening process was conducted by two independent reviewers, both with expertise in the subject area, who assessed the records in a blinded manner for eligibility based on pre-established criteria. In cases of disagreement or uncertainty regarding inclusion, the decision was discussed and resolved by a third reviewer, who was responsible for the final assessment. A detailed summary of all scientometric workflow is provided in Supplementary Table S2. Workflow of the Scientometric Analysis Process.

### 2.9. Scientometric Analyses

**Figure 2:**
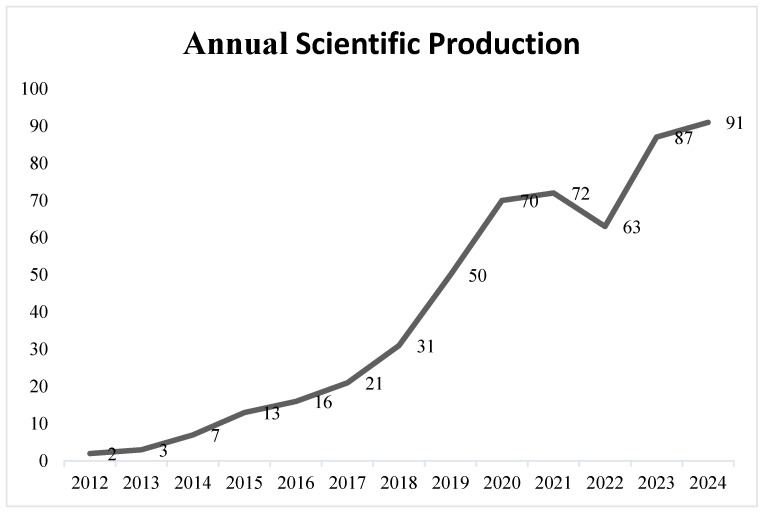
Temporal trend of publications on CrossFit training.

**Figure 3:**
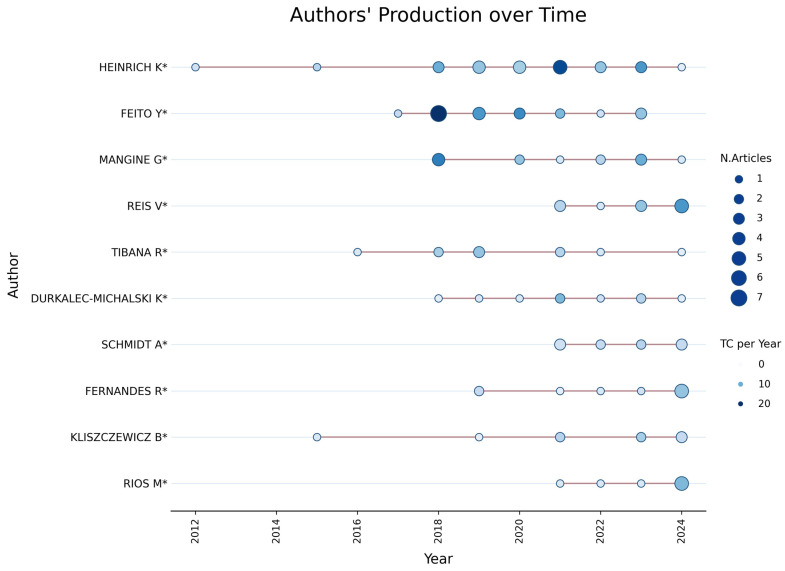
**Scientific production of leading authors between 2012 and 2024.** The bubble chart illustrates the temporal distribution of publications by the most productive authors, where bubble size is proportional to the number of articles published in each year and color intensity represents the annual citation impact of those publications.

**Figure 4:**
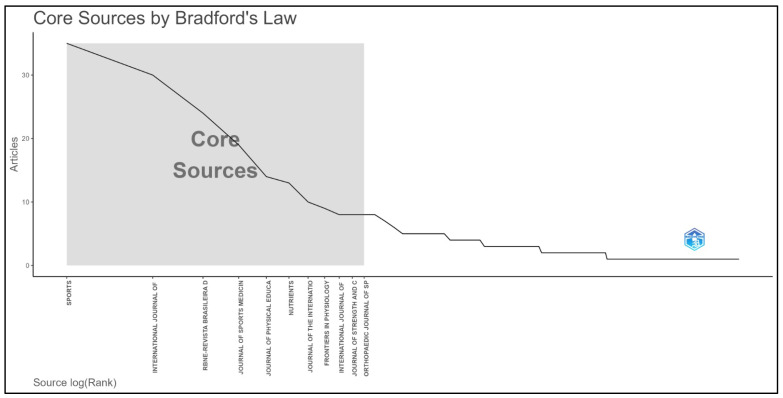
**Core sources according to Bradford’s Law [43].** The figure illustrates the concentration of scientific production within a restricted set of journals that form the core sources of CrossFit^®^ research, while the remaining publications are distributed across a broader set of peripheral journals.

**Figure 5:**
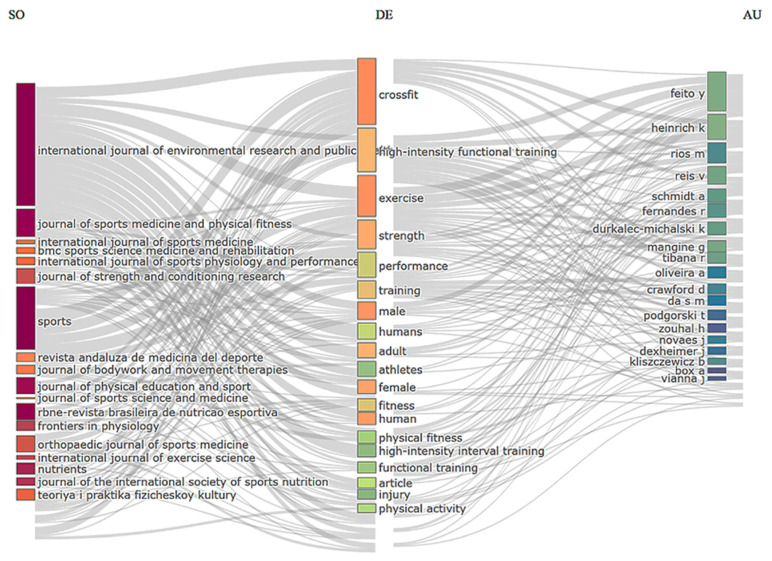
**Sankey diagram illustrating the publication network in CrossFit^®^ research**. The map highlights the relationships between the most productive journals publishing on CrossFit^®^ training, the most frequently used descriptors (keywords), and the most productive authors. Legend: SO—Sources (Journals); DE—Descriptors (Keywords); AU—Authors.

**Figure 6:**
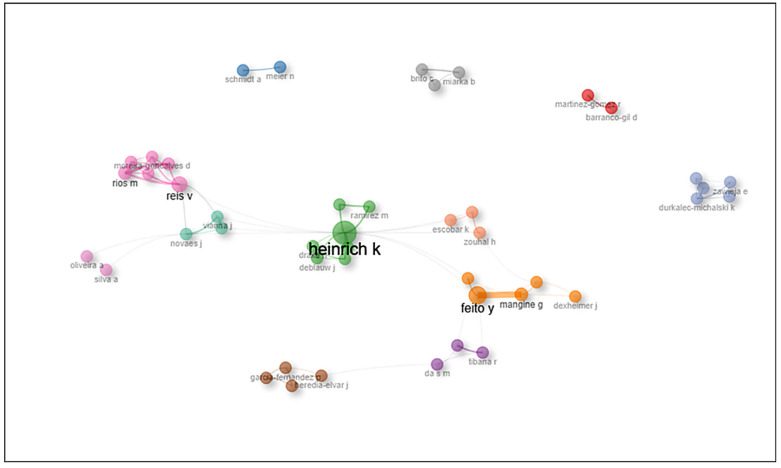
**Co-authorship network among researchers investigating CrossFit^®^ training.** Illustrates the co-authorship network among the main researchers in the field of CrossFit^®^ training. Each node represents an author, and node size reflects the volume of scientific production. The colors differentiate collaboration clusters, while the connecting lines represent co-authorship relationships established between researchers.

**Figure 7:**
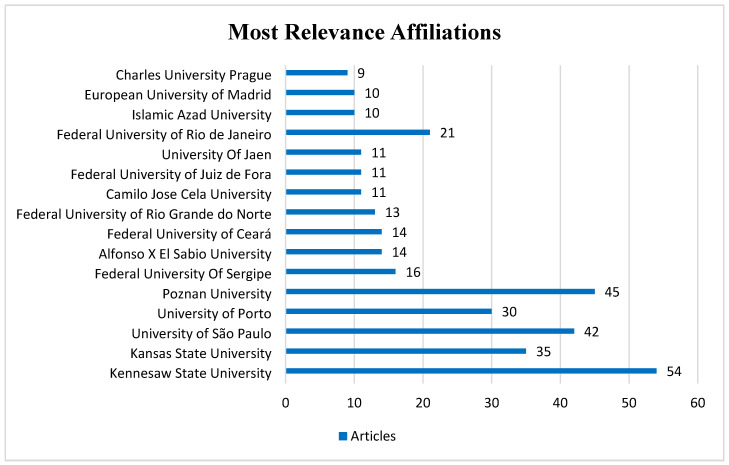
**Institutional Scientific Production in CrossFit^®^ Research.** The bar chart displays the institutions with the highest number of publications in the dataset. The horizontal axis represents the number of articles, while the vertical axis lists the institutional affiliations of the authors.

**Figure 8:**
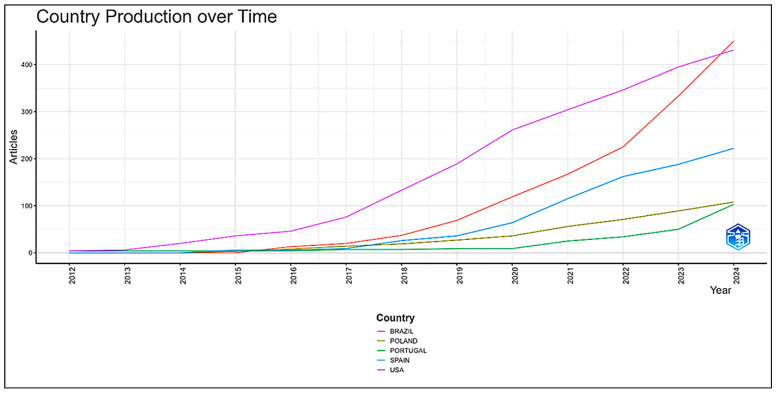
**Country production over time in CrossFit^®^ research.** The line graph illustrates the temporal evolution of scientific output across the most productive countries. The horizontal axis represents the years analyzed (2012–2024), while the vertical axis shows the cumulative number of publications.

**Figure 9:**
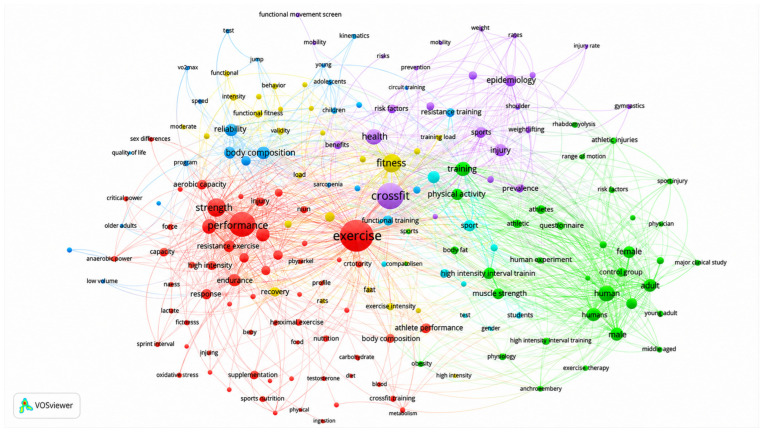
**Keyword co-occurrence network.** The map illustrates the thematic organization of the literature through clusters of frequently associated keywords. Node size reflects the frequency of keyword occurrence, while the connecting lines indicate the strength of the relationships between terms.

**Figure 10:**
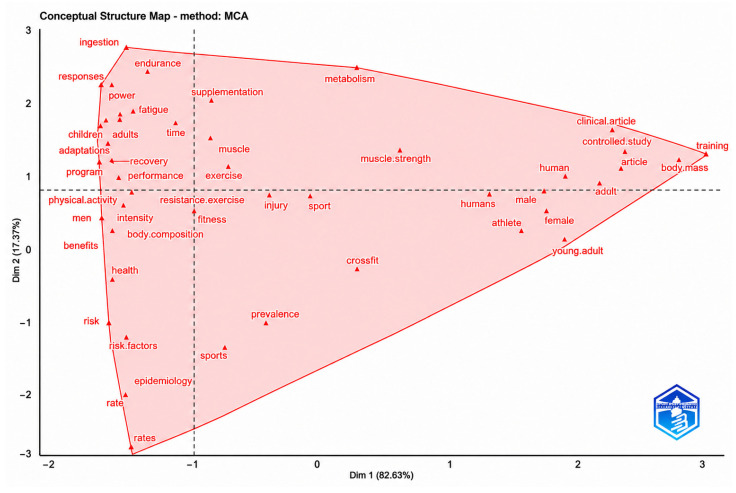
**Conceptual Structure Map generated through Multiple Correspondence Analysis (MCA).** The map organizes keywords into two main dimensions: the horizontal axis (Dimension 1) represents the central thematic proximity between terms, while the vertical axis (Dimension 2) distinguishes secondary variations among conceptual groupings. The shaded polygon connects the most representative concepts, allowing visualization of the conceptual structure of the field.

**Figure 11:**
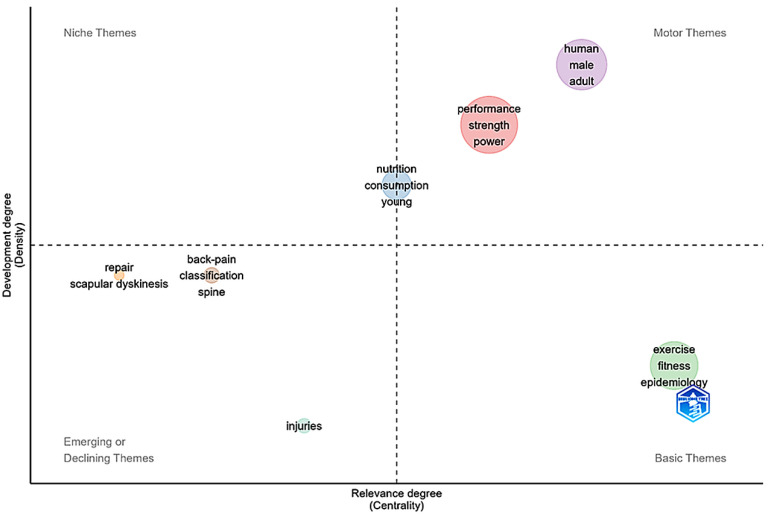
**Thematic map of the scientific literature on CrossFit^®^.** The diagram organizes themes into four quadrants according to their centrality (relevance to the field) and density (degree of development): (i) motor themes, representing central and highly developed topics; (ii) basic themes, which structure the conceptual foundation of the field; (iii) niche themes, representing specialized and well-developed topics with limited centrality; and (iv) emerging or declining themes, which correspond to topics that are either in early development or losing relevance.

**Figure 12:**
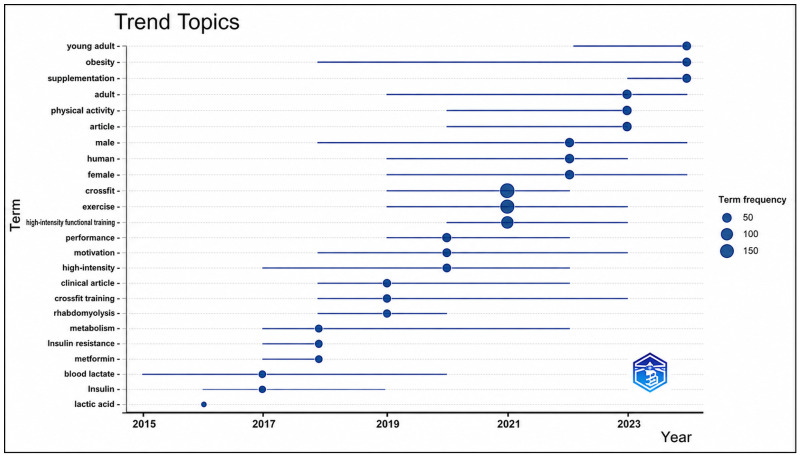
**Trending topics in CrossFit^®^ research.** The graph illustrates the temporal evolution of the main keywords used in the literature. Horizontal lines represent the duration during which each term appears in the scientific production, while the size of the circles indicates the frequency of occurrence of the respective keywords.

Scientometric analyses were conducted using Bibliometrix (version 4.3.2) implemented in R (version 4.4.2) [28]; R Core Team, 2024). Additional data organization and graphical preparation were performed using Microsoft Excel (version 2006: Microsoft Corporation, Redmond, WA, USA), while network visualizations were generated using VOSviewer (version 1.6.20) [29]. The integration of these tools enabled comprehensive quantitative mapping of the scientific structure, collaboration networks, and thematic evolution of CrossFit^®^ research. The following metrics and visualizations were generated:

1. Temporal trend of scientific production: The annual evolution of publications was illustrated through a graph generated in Microsoft Excel, presenting the absolute number of articles published during the analyzed period (Figure 2).
2. Author productivity: A ranking of the most productive authors was established based on the total number of publications indexed in the dataset, allowing identification of the leading contributors to CrossFit^®^ research (Figure 3).
3. Most influential journals: Journal distribution was evaluated according to Bradford’s Law, enabling identification of the core journals that concentrate the largest proportion of publications on the topic (Figure 4).
4. Publication network (Three-Field Plot—Sankey diagram): A three-field plot was generated using the metadata fields Sources (SO), Author Keywords (DE), and Authors (AU), illustrating the relationships between journals, thematic descriptors, and leading authors within the field (Figure 5).
5. Most cited documents: The most influential publications were identified and organized in a table including first author, journal, total citations, local citations within the dataset, normalized citations per year, and additional citation-based impact indicators (Table 1).
6. Co-authorship network: Collaboration patterns among researchers were examined through a co-authorship network generated in VOSviewer (v1.6.20). In this visualization, node size represents author productivity, while link strength reflects the intensity of collaborative relationships between authors (Figure 6).
7. Institutional productivity: Institutional contributions were evaluated by analyzing the number of publications associated with each affiliation, allowing identification of the most productive research institutions in the field (Figure 7).
8. Country-level scientific production: The temporal evolution of scientific output across countries was examined to identify geographic patterns and leading nations in CrossFit^®^ research (Figure 8).
9. Conceptual structure and thematic trends: (i) Keyword co-occurrence network: constructed using Author Keywords, enabling identification of the main thematic clusters in the literature (Figure 9). (ii) Conceptual Structure Map (Multiple Correspondence Analysis—MCA): A two-dimensional conceptual map was generated to represent the relationships between keywords. Dimension 1 reflects the central thematic proximity among terms, whereas Dimension 2 distinguishes secondary conceptual variations among thematic clusters (Figure 10). (iii) Thematic map: This analysis classified research themes into four quadrants (motor themes, niche themes, emerging or declining themes, and basic themes), according to their centrality and density within the scientific field (Figure 11). (iv) Trending topics: The 25 most frequent keywords across the entire study period were identified automatically using Bibliometrix, allowing the detection of emerging and rapidly expanding research topics. (v) Thematic evolution (Sankey diagram): A Sankey-based visualization was used to illustrate the temporal transition of research themes across two automatically defined periods (2012–2021 and 2022–2024) (Figure 12).

A detailed summary of all scientometric analyses, their objectives, and the software used is provided in Supplementary Table S3. Overview of Scientometric Analyses, Objectives, and Software Used. 

## 3. Results and Discussion

The analysis covered the period from September 2012 to November 2024, comprising 526 documents distributed across 226 academic journals, thereby indicating a broad dispersion of CrossFit^®^ research across scientific outlets. The dataset revealed an average annual growth rate of 37.4% highlighting the rapid expansion of scientific interest in this modality. This growth reflects the increasing academic attention devoted to CrossFit^®^ training and its physiological, biomechanical, and health-related implications. Although often situated within the broader context of HIFT, the present findings specifically capture the expansion of CrossFit^®^-centered research.

Although a formal characterization of study designs was beyond the scope of the present scientometric analysis, previous reviews in the field have described CrossFit^®^ research as being largely composed of observational and short-term experimental investigations, with comparatively fewer longitudinal and controlled intervention studies [24,30,33,34,35]. In this context, the rapid expansion in publication volume observed over recent years should be interpreted cautiously, as quantitative growth does not necessarily imply proportional methodological diversification or conceptual consolidation. Therefore, future investigations may benefit from greater emphasis on longitudinal monitoring, controlled interventions, and integrative research designs capable of better reflecting the complexity and ecological dynamics of CrossFit^®^ practice.

However, this expansion should be interpreted within the context of broader structural transformations in sport science and the scientific publishing landscape. Over the past decade, the consolidation of high-intensity training modalities, particularly within training modalities conceptually aligned with CrossFit^®^, often described in the literature under the broader framework of HIFT, has driven a substantial increase in experimental and applied research focused on performance optimization and physiological adaptations [25,32,36,37]. Concurrently, the expansion of open-access publishing models and the growing visibility of multidisciplinary journals indexed in major databases have contributed to the overall rise in sport and exercise science publications [38,39]. This trend has been described as part of a broader “publication inflation” process, in which emerging and highly applied topics, such as CrossFit^®^, experience accelerated scientific dissemination due to their strong practical relevance and global popularity.

Thus, the observed growth in CrossFit^®^ research should not be interpreted solely as a field-specific phenomenon, but rather as the result of an interaction between the expansion of the modality itself and wider changes in the scientific ecosystem. The mean age of the documents was 4.16 years, indicating a predominance of recent publications and suggesting that the field is still in a relatively early stage of scientific consolidation. On average, each publication received 12.4 citations, reflecting a moderate but increasing level of scientific impact.

Regarding authorship patterns, a total of 2322 researchers contributed to the publications included in the dataset. Only 11 articles were single-authored, while the majority resulted from collaborative efforts. The average of 5.73 co-authors per document reinforces the multidisciplinary nature of the field, encompassing areas such as sport science, exercise physiology, and performance research. International collaboration was observed in 18.2% of the publications, indicating that, despite the global visibility of CrossFit^®^ research, the proportion of international partnerships remains relatively modest compared to other rapidly expanding areas within sport science. This finding suggests opportunities to strengthen global research networks and foster broader international collaboration in future studies. The relatively modest rate of international collaboration suggests that, despite global dissemination of the modality, research networks remain partially fragmented, which may limit the development of large-scale, multicenter, and methodologically robust investigations.

### 3.1. Temporal Trend of Scientific Production

Figure 2 presents the quantitative evolution of scientific production related to CrossFit^®^ between 2012 and 2024, revealing a consistent upward trend in the number of publications, particularly from 2018 onward. This pattern reflects the progressive consolidation of CrossFit^®^ as a topic of scientific interest within exercise science and sports performance research. Between 2021 and 2024, the number of publications increased by 28.1%, reaching a peak of 91 publications in 2024, which reinforces the recent expansion of research dedicated to CrossFit^®^ training and related HIFT-based conceptual frameworks.

Across the entire period analyzed, from 2012 to 2024, a total growth of 4450% in scientific output was observed, with the number of publications rising from only two studies in 2012 to 91 in 2024. This trajectory corresponds to an average annual growth rate of approximately 37.4%, highlighting the rapid development of the field and the growing academic interest in investigating the physiological, biomechanical, performance, and health-related aspects associated with CrossFit^®^ practice. This substantial increase in publication volume also suggests that CrossFit^®^ research is suggesting increasing diversification and stabilization of scientific production over time, characterized by expanding research networks and diversification of thematic approaches.

While the rapid expansion of CrossFit^®^-related publications reflects increasing scientific and practical interest in the modality, this growth should be interpreted with caution. Accelerated publication rates do not necessarily correspond to proportional advances in methodological rigor or theoretical development. Similar patterns have been observed in other rapidly expanding areas of sport science, where the proliferation of studies is often accompanied by heterogeneity in study design, limited sample sizes, and the expansion of publication volume should not necessarily be interpreted as proportional advancement in methodological complexity or conceptual integration.

In this context, the current scientometric findings suggest that, although the volume of research has increased substantially, the field may still be in a phase of consolidation, in which descriptive and exploratory studies predominate over robust experimental and longitudinal designs. This imbalance highlights the need to critically interpret the expansion of the literature, distinguishing between quantitative growth and qualitative maturation of the field.

The more pronounced growth in scientific production between 2018 and 2024 can be partly explained by the increasing global popularity of CrossFit^®^, the expansion of training facilities, and the growing number of practitioners worldwide. Although estimating the total number of participants remains challenging, large-scale events such as the CrossFit Open have reported more than 415,000 registered participants in a single year [40]. Additionally, it is estimated that the United States hosts more than 7300 affiliated CrossFit^®^ gyms (“boxes”), followed by Brazil with approximately 1500 affiliated facilities [11,12]. This expansion has contributed to consolidating CrossFit^®^ not only as a competitive sport but also as a widely adopted conditioning strategy within the fitness industry [13,14,15,16].

#### 3.1.1. Leading Authors and Production over Time

Figure 3 illustrates the scientific production of the leading authors in CrossFit^®^ research between 2012 and 2024. Among them, Heinrich K. and Feito Y. stand out as the most productive researchers, each contributing seven publications, with continuous activity since approximately 2016. Such continuity in publication output suggests the consolidation of stable research agendas and sustained scholarly engagement within the field, a pattern commonly observed in the maturation of emerging scientific domains [41,42].

Following these authors, Mangine G. contributed five publications, while Reis V. and Tibana R. each produced four publications, highlighting the active participation of Brazilian researchers in the international development of CrossFit^®^ research. Overall, the temporal distribution of author productivity indicates a transition from an initial phase characterized by low publication volume and strong concentration of output among a limited number of researchers (2012–2016) to a more diversified and internationally distributed scientific network between 2018 and 2024. Within this evolving landscape, Heinrich and Feito emerge as structurally influential contributors to the field, while the entry of additional researchers after 2020 reflects the expansion and renewal of scientific productivity related to CrossFit^®^ training.

This concentration of productivity among a limited number of authors may also indicate a degree of intellectual centralization within the field, potentially influencing research agendas and thematic priorities. While such leadership is typical in emerging domains, it may also limit conceptual diversity if not accompanied by broader international collaboration.

#### 3.1.2. Most Influential Scientific Journals

Figure 4 shows a classic Bradford-type distribution, in which a relatively small core of journals concentrates a substantial share of the publications on CrossFit^®^, while a long tail of peripheral journals reflects the dispersion of the remaining output across multiple outlets. This pattern is consistent with the progressive organization of an emerging field, in which a nuclear set of journals becomes the main channel for scientific communication while other journals absorb more specialized or interdisciplinary contributions [43,44].

Among the identified sources, *Sports* emerged as the primary dissemination channel for CrossFit^®^ research, presenting the highest publication volume and occupying a central position within the core zone. This prominence is consistent with the journal’s focus on sport and exercise science and its strong visibility in major international databases. According to official metrics, *Sports* reported a 2024 Impact Factor of 2.9, a 5-year Impact Factor of 3.3, and a Q1 ranking in “Sport Sciences.” Published by MDPI and widely indexed, its centrality appears to reflect both thematic alignment and high editorial visibility, enhancing its attractiveness for studies on performance, training, and sport-related interventions.

The *Revista Brasileira de Nutrição Esportiva* (RBNE) also plays a relevant role within the network. Although it does not share the same international bibliometric prominence as leading journals, its presence highlights its importance in disseminating research at the interface of CrossFit^®^, sports nutrition, supplementation, and applied exercise physiology. Its open-access model and accessibility, particularly within Portuguese-speaking contexts, likely facilitate the dissemination of applied studies, especially in areas such as ergogenic strategies and body composition.

The *Journal of Sports Medicine and Physical Fitness* appears as another relevant outlet, consistent with its established focus on sports medicine, physical conditioning, injury, and performance. Its presence reinforces the clinical and performance-oriented dimension of CrossFit^®^ research, even if it is less central in terms of publication volume compared to *Sports*. Similarly, the *Journal of Physical Education (Maringá)* contributes to the diversification of the publication landscape by providing a broader disciplinary platform encompassing physical education, health, and leisure. Its continuous publication model, open-access policy, bilingual publication, and alignment with open-science principles make it particularly accessible to Latin American researchers, which may explain its inclusion in the network.

The presence of journals with lower publication frequency suggests both thematic diversification and a degree of dispersion, a common feature of fields still undergoing consolidation [45]. In the case of CrossFit^®^, this dispersion likely reflects its multidimensional nature, spanning sport science, strength and conditioning, rehabilitation, nutrition, public health, and physical education.

The *International Journal of Environmental Research and Public Health* (IJERPH), also published by MDPI, further illustrates this expansion. With a broad transdisciplinary scope and a 2024 CiteScore of 8.5 (Q1 across multiple categories), its presence indicates that part of the CrossFit^®^ literature has moved beyond performance-focused discussions to incorporate health promotion, behavioral outcomes, and population-level perspectives.

Compared with the bibliometric study “Evidence Provided by the Literature on the Use of CrossFit Elements in Sport,” the present findings are partially convergent. Both analyses identify a rapid increase in publications and the relevance of a limited set of influential journals and authors [26]. However, by integrating multiple databases, the present study reveals a more heterogeneous journal ecosystem and a stronger interdisciplinary profile of CrossFit^®^ research, in contrast to studies restricted to a single database.

Overall, the publication network is characterized by a relatively small core of influential journals alongside a broader dispersion across diverse outlets. This pattern reflects the hybrid and evolving nature of the field, with contributions spanning performance, health, nutrition, education, and sports medicine.

### 3.2. Publication Network in CrossFit^®^ Research

Figure 5 shows the Sankey diagram displaying the network in CrossFit^®^. The left column highlights, in red, the most productive journals publishing on CrossFit^®^ research. Among the leading outlets identified in the dataset are Sports, International Journal of Environmental Research and Public Health, Journal of Sports Medicine and Physical Fitness, Revista Brasileira de Nutrição Esportiva, and Journal of Physical Education and Sport.

The convergence between the Bradford distribution and the Sankey visualization reinforces the consistency of the core dissemination structure, indicating that a relatively small group of journals concentrates both the publication volume and the conceptual circulation of CrossFit^®^ research. Similar patterns have been reported in previous bibliometric analyses examining the scientific literature on CrossFit^®^ and high-intensity functional training, which also identified a restricted group of journals responsible for a large proportion of publications in the field.

In the central column of the diagram (Descriptors—DE), the most frequent keywords include “CrossFit”, “exercise”, “strength”, “performance”, and “training”, reflecting the central research focus on physiological responses, neuromuscular adaptations, and performance outcomes associated with CrossFit^®^ training. Within the scientific literature, CrossFit^®^ is often described using the broader term HIFT, which functions as a generalized and academically standardized descriptor for training modalities involving high-intensity, functional movements. This practice is partly related to the fact that CrossFit^®^ is a registered trademark, leading some authors to adopt more generic terminology when describing similar training structures. Consequently, HIFT terminology appears in several influential studies investigating physiological and performance-related responses to CrossFit^®^-like protocols [32,46]. However, it is important to emphasize that the present analysis is based exclusively on CrossFit^®^-specific studies, as defined by the inclusion criteria adopted in this study. Therefore, the presence of HIFT-related terminology in the network should be interpreted as a reflection of terminological conventions within the literature, rather than as an indication that the dataset encompasses the broader spectrum of functional training modalities.

Additional descriptors frequently observed in the network include terms related to sex (“male”, “female”), age groups (“adult”, “athletes”), and components of physical fitness (“fitness”, “physical fitness”). These patterns suggest a methodological tendency to investigate young and physically active populations, which is consistent with previous systematic reviews in the field [32,47].

In the right column of the Sankey diagram (Authors—AU), prominent researchers include Feito Y., Heinrich K., Rios M., and Reis V., who show strong associations with the central descriptors and with the journals occupying the core of the publication network. This interconnection indicates the presence of consolidated research groups that contribute substantially to the development of the field, particularly within North American and Brazilian research institutions, which have played a significant role in advancing the scientific understanding of CrossFit^®^ training.

Finally, the Sankey diagram reveals a high level of interconnectivity between descriptors and journals, suggesting that the scientific literature on CrossFit^®^ increasingly integrates different terminological and conceptual frameworks to describe the modality. This conceptual overlap reflects the hybrid nature of CrossFit^®^, which is simultaneously studied within the contexts of sport performance, strength and conditioning, exercise physiology, and high-intensity functional training methodologies.

### 3.3. Citation Analysis

Table 1 presents the most cited studies on CrossFit^®^ training, highlighting the publications with the greatest impact in the field. The analysis of Total Citations (TC), TC per year, and Normalized TC allows the simultaneous evaluation of both the historical relevance of these studies and their current scientific influence.

**Table 1:** Most cited documents.

Ranking	Paper	DOI	Total Citations	TC per Year	Normalized TC
1	Weisenthal B, 2014, Orthop J Sports Med [48]	10.1177/2325967114531177	161	13.42	4.24
2	Murawska-Cialowicz E, 2015, J Physiol Pharmacol [49]	unidentified	146	13.27	2.24
3	Lichtenstein M, 2016, Addict. Behav. Reports [50]	10.1016/j.abrep.2016.02.002	105	10.50	2.80
4	Butcher S, 2015, Open Access J Sports Med [51]	10.2147/OAJSM.S88265	102	9.27	1.56
5	Bellar D, 2015, Biol Sport [52]	10.5604/20831862.1174771	91	8.27	1.39
6	Eather N, 2016, J Sports Sci [53]	10.1080/02640414.2015.1045925	85	8.50	2.27

The table presents the publications with the highest citation impact in the field, including Digital Object Identifier (DOI), total citation count (TC), citations per year (TC per Year), normalized citation index, and ranking position within the dataset.

The study by Weisenthal et al. [48], published in the Orthopaedic Journal of Sports Medicine, ranks first in citation count and demonstrates strong influence within the literature. This work describes the epidemiology of injuries associated with CrossFit^®^ practice, providing important data related to safety and injury prevention. Such findings have practical implications for improving training prescription and program design in CrossFit^®^ settings. The high number of citations likely reflects both the methodological rigor of the study and its relevance for subsequent research, consolidating it as a central reference in discussions concerning the risk–benefit relationship of CrossFit^®^ training.

In second place, the study by Murawska-Ciałowicz et al. [49], published in the Journal of Physiology and Pharmacology, represents one of the earliest investigations examining neuroendocrine and metabolic responses to CrossFit^®^ training. The authors reported increases in brain-derived neurotrophic factor (BDNF) and improvements in hormonal and oxidative stress parameters. These findings contributed to a better understanding of the potential links between high-intensity functional training and mechanisms of neuroplasticity, cognitive performance, and neurological health, which aligns with evidence suggesting that resistance and high-intensity exercise may play a protective role in the prevention of neurodegenerative diseases.

Subsequently, the manuscript by Lichtenstein et al. [50], published in Addictive Behaviors Reports, introduced a psychosocial perspective on CrossFit^®^ participation. The study suggested that high levels of engagement in the modality may, in some cases, resemble exercise dependence behaviors, thereby opening new research directions related to motivation, adherence, behavioral patterns, and mental health in functional training contexts. This contribution broadened the interdisciplinary scope of CrossFit^®^ research by incorporating behavioral and psychological frameworks.

The work by Butcher et al. [51], published in the Journal of Sports Medicine, contributed to defining physiological and cardiorespiratory responses to CrossFit^®^ training, including parameters related to internal load, VO_2_max, and ventilatory thresholds during functional training protocols. Similarly, the studies by Bellar et al. [52] (Biology of Sport) and Eather et al. [53] (Journal of Sports Sciences) stand out for their applied perspectives. The former examined strength development, body composition, and anabolic hormonal responses, whereas the latter evaluated high-intensity functional training interventions in youth and school populations, demonstrating the potential adaptability of this training approach to different age groups and contexts.

The six most cited studies present an average of 115 citations and 10.9 citations per year, which represents a substantial citation level for a relatively recent research field that began expanding after 2010. From a qualitative perspective, this influential group of publications can be organized into three complementary thematic axes: epidemiological (injury and safety), physiological (training adaptations and performance), and psychological (behavior and engagement). Together, these axes illustrate the interdisciplinary foundation that characterizes the scientific literature on CrossFit^®^, reflecting the early consolidation of the field and the diversity of research approaches that have shaped its development.

### 3.4. Co-Authorship Network Among Researchers Investigating CrossFit^®^ Training

The co-authorship network presented in Figure 6 reveals the scientific collaboration structure among the most active researchers investigating CrossFit^®^ training. The central cluster (green), led by Heinrich K., emerges as the most influential and interconnected group within the network. This author appears to act as a structural bridge (broker) linking different research groups, particularly those led by Reis V. and Feito Y., thereby facilitating the circulation of knowledge between clusters.

The second major cluster (orange) includes Feito Y., Mangine G., Dexheimer J., Tibana R., and Da S. M., whose research is strongly oriented toward physiological responses, performance outcomes, and safety considerations related to CrossFit^®^ training [12,19,54,55,56]. This group represents one of the most productive and internationally visible collaboration networks within the dataset. Another relevant subcluster includes Ramirez M., Draeger J., and Deblau J., which maintains direct collaborative links with Heinrich K. This connection suggests an extension of the central research network, possibly associated with experimental studies and controlled training interventions examining physiological and performance adaptations.

A further prominent cluster is centered around Reis V. (pink), closely connected with Rios M., Vianna J., Novaes J., and Moreira-Gonçalves D. This group represents one of the main clusters composed of Brazilian researchers, with a research focus on exercise physiology [7,20,57,58], cardiovascular control [56,59], and strength training applications within CrossFit^®^ protocols [8,57,60]. Although this cluster shows a stronger regional composition, the presence of collaborative links with international authors indicates the increasing integration of Brazilian researchers into global scientific networks.

Smaller clusters are also visible in the network. For example, Durkalec-Michalski K. and Zwierzaj E. (blue) represent a collaboration group based primarily in Poland, while Martínez-Gómez C. and Barranco-Gil D. (red) appear as another relatively independent research nucleus, likely associated with Spanish institutions. These clusters suggest nationally concentrated research groups that still exhibit limited levels of international collaboration. Additionally, authors such as Schmidt A. and Meier N. (gray) represent small, localized collaborations, reflecting the typical thematic dispersion observed in emerging research fields, where multiple independent research initiatives coexist before stronger collaborative structures become fully established.

Beyond the structural description of collaboration patterns, the configuration of the co-authorship network provides important insights into the dynamics of knowledge production within the field. The concentration of scientific output around a limited number of highly connected authors suggests that research agendas may be partially shaped by these central actors, potentially influencing thematic priorities and methodological approaches. In this sense, the observed network structure reflects not only patterns of collaboration but also underlying asymmetries in scientific influence and knowledge dissemination. Such centralization may accelerate knowledge production in specific domains, particularly those related to performance and physiology, while potentially limiting the diversification of research perspectives. Therefore, expanding collaborative networks and promoting greater international integration may be essential for fostering conceptual innovation and enhancing the overall maturity of CrossFit^®^ research.

### 3.5. Scientific Production by Institutions and Affiliations

The institutional analysis (Figure 7) identifies Kennesaw State University as the leading international research hub in CrossFit^®^ studies, accounting for 15.6% of the total scientific production in the dataset. It is followed by Poznań University of Physical Education with 13.0%, and University of São Paulo (USP) with 12.1% of the publications. This distribution highlights the concentration of research centers in developed countries such as the United States and Poland, while also revealing the significant emergence of research hubs in Latin America, particularly Brazil.

When Brazilian institutions are considered collectively, including Federal University of Rio de Janeiro, Federal University of Ceará, Federal University of Rio Grande do Norte, Federal University of Juiz de Fora, and Federal University of Sergipe, their combined output represents approximately 26.8% of the total sample, demonstrating the strong participation of Brazilian research groups in the development of CrossFit^®^ science.

These findings are consistent with the panorama described by Feito et al. [25], who identified the United States as the origin of approximately 66% of the scientific publications on CrossFit^®^, highlighting the prominent contributions of researchers such as Yuri Feito and Katie Heinrich at Kennesaw State University, which has become a central international hub for research on the modality. Similarly, Stanciu and Voiculescu [26] confirmed Kennesaw State University as the institution with the highest number of publications in their bibliometric dataset, followed by Poznań University of Physical Education, largely driven by the contributions of Murawska-Ciałowicz, and by the University of São Paulo, which stands out as the leading Latin American institution in the field.

Comparatively, bibliometric studies in related areas, such as research on High-Intensity Interval Training (HIIT) in cardiac rehabilitation, have identified a similar structural pattern. For instance, Liu et al. [61] reported that countries such as Norway, Canada, and the United States dominate the field, with institutions such as Norwegian University of Science and Technology and University of Montreal functioning as central research hubs. This convergence suggests that research on high-intensity training modalities, including CrossFit^®^ and other forms of high-intensity functional training, follows a comparable pattern of scientific diffusion.

Thus, the institutional landscape of CrossFit^®^ research reflects a field undergoing global scientific maturation, characterized by consolidated research hubs in the United States, growing European participation, and a notable expansion of Brazilian research groups. This configuration suggests both thematic diversification and the strengthening of international collaboration networks in the study of CrossFit^®^ training.

### 3.6. Temporal Evolution of Scientific Production by Country

The analysis (Figure 8) indicates that the United States maintains a leading position in scientific production related to CrossFit^®^, which is expected given that both the modality and the conceptual foundations of high-intensity functional training originated and were initially disseminated in the country. This predominance is supported by the large number of practitioners, the extensive network of affiliated training centers, and the presence of specialized research laboratories responsible for early epidemiological, physiological, and psychosocial investigations [25,26]. In addition, U.S. leadership reflects broader structural characteristics, including a consolidated research ecosystem, robust funding mechanisms, high institutional density, and early integration between applied training practices and academic research [25,34]. Such a pattern is commonly observed in sport science, where scientific leadership is frequently associated with both cultural origin and research infrastructure.

From 2021 onward, a marked increase in Brazilian scientific production can be observed. This expansion appears to be associated with the strengthening of national research infrastructure and the consolidation of graduate programs in physical education, sport sciences, and human movement sciences. Notable examples include the Graduate Program in Biodynamics of Human Movement at the School of Physical Education and Sport of the University of São Paulo and the Graduate Program in Movement Sciences at São Paulo State University, both of which have contributed to researcher training and the expansion of experimental studies in exercise physiology and sport performance. Furthermore, the broader expansion of graduate programs in Brazil, supported by national evaluation systems such as CAPES, has likely contributed to increasing research productivity in the field [62].

The growing engagement of the Brazilian scientific community with CrossFit^®^ research also appears to be related to the rapid popularization of the modality in the country, which increased the demand for scientific evidence to support coaching practices, strength and conditioning strategies, and health promotion interventions [63,64,65]. This trend reflects a broader process of scientific growth in emerging research systems, where productivity is influenced by institutional consolidation, international collaboration, and evaluation policies that stimulate publication output. In parallel, the cultural diffusion of CrossFit^®^ in Brazil has likely contributed to shaping research priorities and expanding interest in applied investigations.

Taken together, these findings suggest that the global distribution of CrossFit^®^ research is influenced by the interaction between structural, institutional, and cultural factors. While the early scientific production was strongly associated with countries of origin, subsequent expansion reflects broader processes of scientific diffusion and the progressive integration of emerging research systems into the international literature.

### 3.7. Conceptual Structure and Thematic Trends of the Scientific Field

#### Keyword Co-Occurrence Network

The red cluster, located in the central and lower-left region of the map, highlights the predominant interest in physical performance, body composition, and physiological responses associated with CrossFit^®^ training (Figure 9). Terms such as performance, strength, endurance, resistance exercise, body composition, and recovery indicate a strong concentration of studies focused on exercise physiology, strength training, and metabolic adaptations related to CrossFit^®^ practice [7,20,57,58,60,66,67]. Several investigations within this cluster also refer to the broader framework of HIFT, frequently adopted in the scientific literature as a general descriptor for training modalities sharing characteristics with CrossFit^®^ [32,47,68,69,70]. This cluster therefore represents the central performance- and physiology-oriented domain of the literature. However, the prominence of these themes should be interpreted cautiously, as the inclusion of outcome-oriented descriptors in the search strategy may have amplified the representation of physiology- and performance-related studies within the dataset.

The green cluster, positioned on the right side of the map, concentrates studies related to health outcomes, injury prevention, population characteristics, and human adaptations to CrossFit^®^ practice [15,22,50,71,72,73,74,75]. Keywords such as injury, prevalence, athletes, humans, male, female, adult, and risk factors indicate increasing interest in safety, epidemiology, and health-related outcomes across different participant groups.

The blue cluster represents studies examining physiological and biomechanical dimensions of CrossFit^®^ training, including anaerobic power [58,76,77,78], VO_2_max [51,79,80], mobility [81,82,83], and kinematics [84,85]. These investigations are primarily focused on physical capacities, movement efficiency, and physiological responses associated with high-intensity functional training protocols.

The purple cluster corresponds to epidemiological and behavioral research organized around terms such as epidemiology [86,87,88], questionnaire [89,90], health [16,91,92], prevention [72,83,93], and injury rate [86,87,88]. This group reflects observational studies centered on participant characterization, psychosocial variables, and health-related outcomes.

Finally, the yellow cluster suggests a relatively less explored research area involving youth populations, behavioral factors, and pedagogical applications of CrossFit^®^ training. Keywords such as adolescents [16,94,95], benefits [96,97], quality of life [6,98,99], and behavior [40,100,101] indicate emerging interest in behavioral and educational perspectives within the field.

Overall, the keyword network demonstrates that CrossFit^®^ research is predominantly centered on performance, physiological responses, and injury-related outcomes, whereas psychosocial, pedagogical, and population-specific dimensions remain comparatively less represented. Although the literature shows increasing thematic diversification, the field still appears largely oriented toward performance- and physiology-related outcomes. Future investigations may benefit from greater emphasis on behavioral and population-specific approaches, particularly involving women, older adults, adolescents, and clinical populations.

### 3.8. Conceptual Structure Map (MCA)

The analysis (Figure 10) highlights the conceptual organization of the scientific literature on CrossFit^®^, which is structured around three main thematic axes. The first axis, located in the upper-left quadrant, is associated with physical performance and physiological adaptations to exercise. Keywords such as ingestion, performance, endurance, power, fatigue, program, and adaptations indicate a strong conceptual proximity between studies focused on nutritional, metabolic, physiological, and sport performance variables [18,19,20,51,57,66,102]. This conceptual grouping reflects the predominance of investigations examining physiological responses and biodynamic adaptations associated with CrossFit^®^ practice.

The second axis, located in the lower region of the map, groups terms such as risk, epidemiology, and injury [31,86,87,88,103,104,105]. This thematic domain is conceptually associated with studies addressing injury prevalence, risk factors, and epidemiological monitoring in CrossFit^®^ practitioners. The proximity between these descriptors suggests a cohesive research line centered on safety, injury surveillance, and preventive approaches across different participant populations.

The third axis, located in the right quadrant, encompasses descriptors related to participant characteristics and training contexts. Keywords such as human, male, female, adult, young adult, and training [91,93,96,106,107] indicate conceptual associations with studies involving population characterization, clinical investigations, body composition analyses, and controlled training interventions. This axis reflects the presence of research focused on demographic profiling and comparative analyses across sex, age groups, and physical condition.

Taken together, the conceptual map indicates that the literature is primarily organized around interconnected domains involving physiological performance, epidemiological monitoring, and participant characterization. The spatial distribution of descriptors suggests a conceptual structure in which exercise physiology and performance-related themes occupy a central position, while clinical, demographic, and injury-related topics form complementary conceptual domains within the field.

Nevertheless, some conceptual gaps remain evident, particularly regarding the integration of physiological and psychosocial dimensions, as well as the limited representation of specific populations such as women, older adults, recreational practitioners, and clinical groups. Expanding these thematic connections may contribute to a broader conceptual understanding of the physiological, behavioral, and health-related dimensions associated with CrossFit^®^ training.

### 3.9. Thematic Map

Figure 11 maps the thematic structure of the scientific field according to the dimensions of density (level of development) and centrality (structural importance). The motor themes, located in the upper-right quadrant, are centered around the keyword’s performance, strength, and power, indicating that physiological and performance-related variables remain the most developed and structurally central topics in the literature. These concepts reflect the historical emphasis of the field on muscular adaptations, strength development, and power output associated with CrossFit^®^ training [18,20,57,102]. It is important to note that the prominence of these themes may partially reflect the search strategy adopted in the present study, which included descriptors specifically related to physiological and performance outcomes. Within the same quadrant, the presence of the terms human, male, and adult highlights the predominance of experimental studies involving healthy adult participants and trained athletes [87,90,100].

The basic themes, positioned in the lower-right quadrant, include terms such as exercise, fitness, and epidemiology, which represent central conceptual and methodological components of the field [15,19,86,87,88,104]. These topics encompass studies related to exercise responses, physical fitness, and health-related outcomes associated with high-intensity functional training. The inclusion of epidemiology within this quadrant also suggests increasing research attention toward injury prevalence, safety, and population health.

The niche themes, located in the upper-left quadrant, include topics such as scapular dyskinesis, repair, back pain, and spine, reflecting specialized research lines focused on clinical, biomechanical, and rehabilitation-related aspects [17,92,108,109,110,111,112]. Although these themes demonstrate relatively high internal development, their lower centrality suggests that they remain more peripheral within the overall thematic structure.

Conversely, the emerging or declining themes, located in the lower-left quadrant, include the keyword injuries, which appears in a relatively peripheral position within the thematic map [86,88,103,113]. This distribution may reflect changes in the research focus related to injury surveillance and prevention in functional training modalities.

Overall, the thematic map demonstrates that CrossFit^®^ research is primarily structured around performance- and physiology-related themes, while additional research areas address health outcomes, injury prevention, rehabilitation, and physical fitness. Nutritional aspects also appear as complementary topics associated with studies examining performance, metabolic responses, and body composition [1,20,54,89,114]. Although the literature demonstrates increasing thematic diversification, physiological and performance-oriented investigations remain the dominant components of the field. Future studies may benefit from broader exploration of behavioral, longitudinal, and population-specific approaches to complement the current thematic structure.

### 3.10. Trend Topics

Figure 12 illustrates the temporal evolution of research themes related to CrossFit^®^. The results indicate that terms such as exercise, performance, and CrossFit became established as the most recurrent topics between 2015 and 2017 [102,115,116,117,118]. These descriptors are associated with studies focused on physiological responses, physical performance, internal load monitoring, and adaptations to high-intensity functional training protocols.

From 2019 onward, the emergence of clinical and metabolic themes becomes more evident, including keywords such as *metabolism*, *insulin resistance*, *blood lactate*, *lactic acid*, and *rhabdomyolysis* [22,58,119,120,121]. The appearance of these descriptors indicates increasing research attention toward physiological and biochemical responses associated with CrossFit^®^ training, particularly in studies examining metabolic stress and exercise-related biomarkers.

In more recent years (2021–2024), additional themes related to participant characteristics and behavioral factors became more frequent, including keywords such as *young adult*, *obesity*, *supplementation*, *female*, and *motivation* [34,121,122,123,124,125,126]. These descriptors are associated with studies examining body composition, exercise adherence, nutritional strategies, sex-related differences, and behavioral aspects of training participation.

When analyzing the temporal trajectory across the study period, the literature demonstrates a chronological progression in research focus. Between 2015 and 2018, studies were predominantly centered on physiological responses and performance outcomes. Between 2019 and 2021, clinical and metabolic descriptors became more prominent. From 2022 onward, research topics increasingly included participant-related, nutritional, and behavioral variables.

Overall, the trending topic analysis demonstrates that CrossFit^®^ research has progressively incorporated a broader range of themes over time, while physiological and performance-related investigations have remained consistently central throughout the analyzed period.

### 3.11. Thematic Evolution (Sankey Diagram)

**Figure 13:**
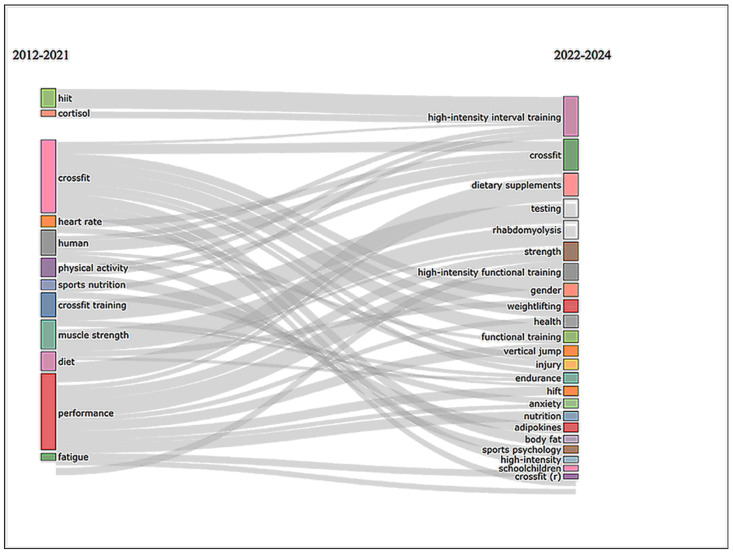
**Thematic evolution of CrossFit^®^ research between two periods (2012–2021 and 2022–2024).** The diagram illustrates how key descriptors from the first period were maintained, transformed, or gave rise to new topics in the subsequent period. The connections between the columns represent the continuity and transition of research priorities across time.

The Figure 13 show the thematic evolution on CrossFit® research, during the initial period (2012–2021), the most recurrent descriptors were strongly concentrated on physiological responses and athletic performance, including terms such as *performance* [19,51,57], *muscle strength* [127,128,129], *fatigue* [78,130,131], and *heart rate* [56,59,103,115]. These descriptors were also accompanied by terms related to acute endocrine and metabolic responses, such as *cortisol* [54,132,133,134] and *diet* [1,17,135,136].

The strong emphasis on variables such as *VO*_2_*max* [19,102,117,131] and *anaerobic power* [52,76,78] reflects the sport-oriented and competitive nature of the modality, during a stage in which the scientific literature was primarily concerned with validating CrossFit^®^ as a training method within exercise physiology and strength and conditioning research.

In the second period (2022–2024), a clear expansion and diversification of research topics can be observed. Although core themes such as CrossFit, strength, and high-intensity functional training remain central to the conceptual structure of the field, new interdisciplinary research axes have emerged. These include clinical and biomedical topics such as *rhabdomyolysis*, *injury*, *adipokines*, and *health* [87,121,137,138,139]. These descriptors reflect the expansion of biomedical and safety-related research, investigating physiological stress responses, inflammatory biomarkers, and injury risk associated with high-intensity functional training.

At the same time, emerging themes such as *anxiety*, *sports psychology*, and *motivation* [18,73,75,116,140] demonstrate the growing incorporation of sport psychology and behavioral sciences into CrossFit^®^ research. These topics address aspects such as exercise adherence, self-efficacy, motivation, and psychological well-being, representing a relatively recent but rapidly expanding area of investigation. This shift suggests a progressive integration between biological and psychological perspectives in understanding the broader health implications of CrossFit^®^ practice.

Another emerging research direction is reflected in descriptors such as *dietary supplements*, *body fat*, and *nutrition* [1,17,21,110,112,124,135]. These themes highlight the strengthening of the interface between exercise physiology and metabolism, demonstrating a growing interest in nutritional strategies aimed at enhancing performance and modulating metabolic responses. This expansion connects CrossFit^®^ research to broader areas such as sports nutrition, endocrinology, and molecular physiology.

Furthermore, descriptors such as *gender*, *young*, *schoolchildren*, and *adult* [79,91,96,107,127,141,142,143] indicate the inclusion of more diverse populations in recent studies, with greater attention to sex differences, age groups, and educational contexts. This demographic expansion reflects the adaptability and accessibility of CrossFit^®^ training, reinforcing its potential applicability across different populations and settings.

Overall, the comparison between the periods 2012–2021 and 2022–2024 indicates that CrossFit^®^ research has evolved from a predominantly physiology- and performance-oriented field toward a more multidimensional domain, integrating perspectives from health sciences, psychology, nutrition, and population-based research. Beyond descriptive trends, these findings indicate an expansion of research themes and increasing inclusion of multidisciplinary perspectives, reflecting a transition from the initial validation of the modality toward a more integrative understanding of its broader health and performance implications.

Despite this progression, an important gap remains between scientific production and practical application. Although much of the literature focuses on physiological and performance outcomes, relatively few studies translate these findings into ecologically valid recommendations for coaches, practitioners, and athletes. Given that CrossFit^®^ is inherently practice-driven, characterized by high variability and real-world complexity, the limited integration between experimental evidence and applied contexts represents a critical limitation. Addressing this gap requires not only more robust study designs but also approaches that incorporate real training environments, practitioner expertise, and longitudinal monitoring. Additional conceptual and applied gaps persist, particularly regarding the integration between physiological, psychological, and ecological dimensions of CrossFit^®^ practice. These issues underscore the need for future research that moves beyond isolated outcome measures, prioritizes longitudinal designs, and strengthens the connection between scientific evidence and applied practice. Such advances are essential for consolidating CrossFit^®^ research as a field capable of both describing the modality and effectively informing its implementation.

From a broader analytical perspective, the findings also reveal structural imbalances in the field’s development. Although the expansion of research topics suggests increasing diversification, the continued dominance of performance-oriented paradigms indicates uneven growth across domains. This asymmetry highlights a gap between the complexity of CrossFit^®^ in practice and the relative simplicity of the research questions most frequently addressed. In this sense, the findings suggest that thematic diversification has progressed more rapidly than the expansion of longitudinal, integrative, or methodologically complex investigations, representing an important challenge for future investigations aiming to strengthen methodological diversity and applied relevance.

## 4. Study Limitations

Despite the methodological rigor and the comprehensive scope of the present scientometric analysis, several limitations should be considered when interpreting the findings. First, the search strategy, although designed to balance sensitivity and specificity, included outcome-oriented descriptors (e.g., performance, strength, VO_2_max, injury, nutrition), which may have introduced a degree of thematic bias. This search string was selected after testing multiple alternative strategies, as it provided the highest number of relevant studies and the most comprehensive dataset for analysis. While this approach maximized sensitivity, it may also have contributed to the predominance of performance- and physiology-related studies observed in the results. Therefore, the thematic structure identified in this study should be interpreted with caution, as it may partially reflect the design and optimization of the search strategy rather than exclusively representing an emergent property of the field.

Second, although the integration of Web of Science, Scopus, and PubMed enhances the comprehensiveness of the dataset, it does not guarantee complete coverage of the literature. Relevant studies published in non-indexed journals, regional databases, conference proceedings, or grey literature may not have been captured. This limitation is particularly relevant in the context of CrossFit^®^ research, where applied and practice-based knowledge may be disseminated outside traditional indexed sources.

Third, the manual screening process, despite being conducted by independent reviewers with a consensus procedure, inherently involves a degree of subjectivity, particularly when distinguishing between CrossFit^®^-specific studies and broader high-intensity functional training (HIFT) research. Although strict inclusion criteria were applied, borderline cases may have influenced the final composition of the dataset.

Fourth, the present study did not include a stratification or assessment of methodological quality or study design. As a result, all publications were treated as equivalent bibliographic units, regardless of their level of evidence, sample size, or experimental rigor. This represents an inherent limitation of scientometric approaches, which focus on patterns of scientific production rather than on the critical appraisal of evidence. Consequently, the findings should not be interpreted as indicators of the strength, validity, or maturity of the evidence base, but rather as representations of the structural organization of the field.

Finally, it is important to recognize that scientometric analyses are limited in their capacity to establish causal relationships or to evaluate the practical applicability of research findings. While the present study provides a comprehensive overview of the evolution, structure, and thematic trends of CrossFit^®^ research, it does not directly assess how this knowledge translates into real-world training practices or clinical applications. Therefore, future research combining scientometric mapping with systematic reviews or meta-analyses is warranted to provide a more integrated understanding of both the structure and the quality of the evidence.

## 5. Conclusions

Through a comprehensive scientometric analysis, this study mapped the scientific production related to CrossFit^®^. The results demonstrate a consistent expansion of the literature over the analyzed period, with a notable peak in publications in 2024. Among the most influential contributors, Heinrich K. emerged as the most productive author and a central actor within the collaboration networks, highlighting his role in structuring the research field. The journal Sports was identified as the main outlet for the dissemination of knowledge on the topic, while the study by Weisenthal et al. [48] appeared as the most cited document, emphasizing the relevance of research focused on injury epidemiology within the modality. In addition, Kennesaw State University was identified as the leading institutional hub for CrossFit research.

Taken together, the present findings indicate that CrossFit^®^ research has experienced rapid expansion, accompanied by increasing diversification of themes and global participation. However, this growth appears to be characterized more by quantitative expansion than by consistent methodological and conceptual consolidation. These findings may indicate limited integration between experimental findings and applied practice within the currently available literature, with comparatively limited emphasis on behavioral factors, ecological validity, and interdisciplinary integration. This imbalance suggests that, despite the growing volume of scientific output, important gaps persist in the integration between research findings and real-world practice. In a modality inherently driven by applied training environments, the limited translation of scientific evidence into ecologically valid recommendations for coaches and practitioners represents a critical challenge.

Advancing the field will require a shift beyond descriptive and exploratory approaches, prioritizing methodological rigor, longitudinal designs, and stronger integration between physiological, psychological, and contextual variables. Such progress may contribute to greater methodological diversification and interdisciplinary integration within CrossFit^®^ research, helping to bridge the gap between experimental findings and the practical demands associated with CrossFit^®^ training and application.

## Data Availability

The datasets generated and analyzed during the current study are available from the corresponding author upon reasonable request.

## Supplementary Materials

The following supporting information can be downloaded at: https://www.mdpi.com/article/10.3390/sports14050213/s1, Supplementary Table S1 (search strategies used across databases), Supplementary Table S2 (workflow of the scientometric analysis process), and Supplementary Table S3 (overview of scientometric analyses and software used).
