# Supplementary material for: From Performance to Health: A Global Scientometric Analysis of the Evolution of CrossFit Research
Source: Sports (Basel). 2026 May 21;14(5):213. doi: 10.3390/sports14050213 (PMC13210993; doi:10.3390/sports14050213)
Supplement: Supplementary file 1 [file sports-14-00213-s001.zip › sports-4287184-supplementary.pdf]

Supplementary Material

Supplementary Table S1. Search Strategies Used Across Databases

("CrossFit" OR "WOD" OR "HIFT" OR "functional fitness") AND ("performance" OR "strength" OR "VO2max" OR "anaerobic" OR "nutrition" OR "diet" OR "supplement" OR "injury" OR "biomechanics" OR "motivation")

Description of search strings, searched fields, and export formats of bibliographic platforms

Standardized Search	Database	File Format
TS=("CrossFit" OR "WOD" OR "HIFT" OR "functional fitness") AND TS=("performance" OR "strength" OR "VO2max" OR "anaerobic" OR "nutrition" OR "diet" OR "supplement" OR "injury" OR "biomechanics" OR "motivation")	WOS	BIB
("CrossFit"[All Fields] OR "WOD"[All Fields] OR "HIFT"[All Fields] OR "functional fitness"[All Fields]) AND ("performance"[All Fields] OR "strength"[All Fields] OR "VO2max"[All Fields] OR "anaerobic"[All Fields] OR "nutrition"[All Fields] OR "diet"[All Fields] OR "supplement"[All Fields] OR "injury"[All Fields] OR "biomechanics"[All Fields] OR "motivation"[All Fields])	PubMed	TXT
TITLE-ABS-KEY("CrossFit" OR "WOD" OR "HIFT" OR "functional fitness") AND TITLE-ABS-KEY("performance" OR "strength" OR "VO2max" OR "anaerobic" OR	Scopus	BIB

"nutrition" OR "diet" OR "supplement" OR "injury" OR "biomechanics" OR "motivation")		

Supplementary Table S2. Workflow of the Scientometric Analysis Process

**Sciometric Review**	Analysis of original articles
**Time frame**	All publications up to December 2024
**Search Strategy**	Identification of descriptors + Boolean operators<br>Search String Construction
**Data collection and file type**	Pubmed = .TXT<br>Scopus =.BIB<br>Web of Science =. BIB
**Integration of files into a single database**	(software R 4.4.2 (R Core Team, 2024)
**Automatic filtering of non-original documents**	Bibliometrix, version 4.3.2<br>(Removal of systematic reviews, narratives, meta-analyses, letters to the editor, chapters, conferences, and gray literature.)
**Manual screening**	Removal of duplicates and remaining reviews<br>Scope screening<br>(Inclusion: original articles, regardless of language and related to the topic)<br>(exclusion: systematic reviews, meta-analyses, narratives, letters to the editor, books, book chapters, conference abstracts, and gray literature)
**Sciometric Analysis**	Bibliometrix 4.3.2, implemented in R 4.4.2 (R Core Team, 2024), Microsoft Excel® (Microsoft 365 / Office), and VOSviewer 1.0.0

Supplementary Table S3 Overview of Scientometric Analyses, Objectives, and Software Used

Analysis	Objective	Software Used
**Annual Scientific Production (Temporal Trend)**	To quantify the yearly evolution of publications and identify growth patterns over time.	Excel®; Bibliometrix (R)
**Most Productive Authors**	To identify authors with the highest publication output and their temporal productivity patterns.	Bibliometrix (R)
**Leading Journals (Bradford's Law)**	To determine the core journals in the field and classify sources according to Bradford's distribution.	Bibliometrix (R)
**Three-Field Plot (Sankey: Authors × Keywords × Sources)**	To visualize the relationship between authors, keywords, and journals.	Bibliometrix (R)
**Most Cited Documents**	To identify documents with the highest impact based on total, normalized, and annual citations.	Bibliometrix (R); Excel®
**Co-authorship Network**	To map collaboration patterns among researchers and identify influential authors.	VOSviewer
**Institutional Productivity**	To quantify the scientific contribution of institutions in absolute publication numbers.	Bibliometrix (R)
**Country Scientific Production Over Time**	To evaluate geographic distribution and temporal evolution of scientific output by country.	Bibliometrix (R)
**Keyword Co-occurrence Network**	To identify thematic clusters and conceptual relationships through keyword frequency and co-occurrence.	VOSviewer; Bibliometrix (R)
**Conceptual Structure Map (MCA)**	To analyze conceptual organization of the field and identify primary and secondary thematic dimensions.	Bibliometrix (R)
**Thematic Map**	To classify themes according to centrality and density (emerging, basic, niche, and motor themes).	Bibliometrix (R)
**Trending Topics**	To identify the most frequent and emerging terms over time.	Bibliometrix (R)
**Temporal Evolution (Sankey Diagram)**	To visualize how topics evolve and transition across time periods.	Bibliometrix (R)
